# Algorithmic gaze annotation for mobile eye-tracking

**DOI:** 10.3758/s13428-025-02803-2

**Published:** 2025-09-17

**Authors:** Daniel Mueller, David Mann

**Affiliations:** Department of Human Movement Sciences, Faculty of Behaviour and Movement Sciences, Amsterdam Movement Sciences and Institute Brain and Behavior Amsterdam (iBBA), Vrije Universiteit Amsterdam, Amsterdam, The Netherlands

**Keywords:** Eye-tracking, Gaze analysis, Human interactions

## Abstract

Mobile eye-tracking is increasingly used to study human behavior in situ; however, the analysis of the footage is typically performed manually and therefore is slow and laborious. The aim of this study was to examine the extent to which the footage obtained using mobile eye-tracking could be annotated automatically using computer vision algorithms. We developed an open-source Python package that combined two computer vision algorithms to automatically annotate human-body-related areas of interest when two participants interacted with each other. To validate the algorithm, three experienced human raters coded the gaze direction with respect to one of seven a priori defined areas of interest during the task. To test the reliability of the algorithm, the agreement between the human raters was compared with the results obtained from the algorithm. A total of 1,188 frames from 13 trials were compared, with the results revealing substantial agreement between the algorithm and human raters (Krippendorff’s alpha = 0.61). The algorithm strictly annotated whether gaze was within or outside of the specified areas of interest, whereas human raters seemed to apply a tolerance when gaze was lying slightly outside the areas of interest. In sum, the computer algorithmic approach appears to provide a valid means of automatically annotating mobile eye-tracking footage in highly dynamic contexts. The possibility of automatically annotating eye-tracking footage of human interactions allows for automatic assessment of visual attention, gaze, and intentions across sectors such as educational settings, pedestrian navigation, and sport.

## Introduction

Mobile eye-tracking plays an increasingly important role in understanding the nature of human interactions. Recent developments in wearable eye-tracking devices have lowered the technical entry barrier for conducting eye-tracking research, with systems being more affordable and user-friendly (Kredel et al., 2017). Eye-tracking technology is used across applied domains to help understand human interactions. We would like to present three examples of human interactions where eye-tracking can have a practical impact. First, in educational settings, eye-tracking measures can be used to understand how experienced teachers interact with students (McDonic et al., 2025). In physical education settings in particular, teachers observe the behaviors and movements of students and are required to provide feedback to the students. Eye-tracking studies enable a deeper understanding to improve feedback processes in educational settings. Second, another application of eye-tracking is pedestrian road safety. For instance, research has been conducted to understand how pedestrians use gaze to navigate and how cues from body language can help understand the intentions for navigation in others (Ridel et al., 2018). Eye-tracking holds promise to enhance our understanding of how humans interact in pedestrian situations, such as a car driver observing pedestrians crossing the road to avoid accidents. Third, a large body of research highlights that expert behaviors across domains are underpinned by specific gaze strategies, with the most prominent cases derived in sport anticipation tasks (Cañal-Bruland & Mann, 2024). For instance, expert goalkeepers in sports perform fast eye movements to process the relevant information from a penalty-taker during run-up. It has been shown that experts perform specific transitions between different limbs that help them anticipate the penalty correctly (Savelsbergh et al., 2002). Studies of expert behavior provide insights into what perceptual information humans rely on when interacting with other humans. The challenge of eye-tracking studies in applied domains is that most tasks are highly dynamic and are thus challenging to analyze in a controlled fashion.

While the data collected using traditional head-restricted eye-tracking with a chin-rest can be analyzed automatically with predetermined areas of interest, the data collected from mobile eye-tracking is predominantly performed manually on a frame-by-frame basis (Macdonald & Tatler, 2018; Tatler et al., 2011). Not only is this manual annotation laborious and tedious, but the results can be highly subjective (Hooge et al., 2018). A more automated approach that allows for reproducible results is, therefore, highly desirable.

The main challenge for mobile eye-tracking in a head-free setup is that the stimulus being viewed is not standardized. The eyes, the head, and even the body of the subject can move freely, as opposed to when viewing a screen from a chin-rest, where the movement of the head is restricted (Valtakari et al., 2021). Consequently, the areas of interest are changing in both shape and location dynamically (Hessels et al., 2018). In studies of human interactions, this challenge is particularly pertinent because the participant wearing the eye tracker interacts with another human who can move freely in the environment and of their own accord. The areas of interest are different for each participant as a result of the participant’s movements. The challenges are manifested prominently in studies conducted in the field of sports, where human interactions are fast-paced and dynamic (Janssen et al., 2023). Because of the movement involved, the frame of reference for detecting areas of interest is continuously changing, and areas of interest can become temporarily occluded due to the movements. As a consequence, the vast majority of published studies continue to manually analyze the eye-tracking footage (Kredel et al., 2023). For eye-tracking studies that are interested in analyzing aspects of gaze fixations, recent developments in eye-tracking software have mitigated the time that is required to analyze the footage on a frame-by-frame basis through the use of fixation-detection algorithms. Both commercial software (Tobii Pro Lab) and open-source solutions (GazeCode) can be used to annotate eye-tracking footage (Benjamins et al., 2018).

To examine human interactions, especially in the context of highly dynamic environments such as those that occur in sports, gaze analysis often involves the manual annotation of footage taken from the scene camera of an eye tracker with a superimposed cursor indicating the direction of gaze. In this context, the rater decides whether gaze was directed towards any of a number of predefined areas of interest. If gaze is directed outside of the areas of interest, then the direction of gaze is typically annotated as being directed towards a miscellaneous direction (e.g., “other”). If gaze is directed towards an area of interest, the rater has to specify which area of interest (e.g., an arm or leg) gaze was directed towards. However, it remains surprisingly challenging to reliably determine where gaze is directed when looking towards another person. The human body is commonly divided into smaller areas of interest. For instance, Williams and Elliott (1999) used six areas of interest (head, chest, pelvis, legs, arms, shoulder) to analyze human interactions in karate. Similar approaches have been used in other combat sports (Hausegger et al., 2019; Milazzo et al., 2016; Piras et al., 2014; Ripoll et al., 1995). This approach is also used in other sports; Singer et al. (1996), for instance, analyzed tennis serves and predefined seven areas of interest (head, shoulders, wrist, knee, racket, and ball, and other). The problem at hand for the manual rater is that there is ambiguity when it comes to distinguishing between the areas of interest. The ambiguity arises, on the one hand, from the subjective interpretation of a manual rater when it comes to annotating footage to a set of areas of interest. For example, the boundary between the hip and chest areas is challenging to interpret from the eye-tracking footage. Also, since the areas of interest are dynamic, the areas can overlap when, for example, an arm is moved in front of the chest, resulting in a temporal spillover of the gaze intersection until the occlusion stops. On the other hand, when manual raters are annotating footage, it remains challenging to keep the annotation quality consistent over a longer period of time. Both of these problems manifest in such a way that when multiple human raters are required to rate the same footage, there is considerable disagreement between even expert raters (Hooge et al., 2018).

Newly developed computer vision algorithms hold promise as a means of automatically identifying areas of interest to improve the consistency of annotations. Specifically, two classes of algorithms are especially well suited for annotating eye-tracking data. The first class consists of *semantic segmentation* algorithms that identify areas of interest in an image on a pixel-by-pixel basis. For eye-tracking, semantic algorithms can be used, for instance, to identify the cornea in order to compute a gaze vector (Chaudhary et al., 2019; Feng et al., 2022) and, more relevant to our study, to detect objects in the field of view (Deane et al., 2022). The segmentation returns a binary map for each pixel in an image by indicating whether it belongs to a specific area of interest. For instance, Deane et al. (2022) used a semantic segmentation algorithm in natural navigation tasks and were able to automatically analyze areas of interest (a person, car, truck, bus) from eye-tracking data. A similar technique was used by Jongerius et al. (2021) in a study using eye-tracking to identify the intersection of gaze with the face of a person positioned in front of the participant. Here, the participant’s gaze behavior was assessed during patient–physician consultation sessions. A computer vision algorithm was used to automatically create a rectangular area of interest that was mapped around the face, which in turn was used to check whether the gaze location was within the rectangular area associated with the face. The performance of the automated tracking, compared to that of human raters, was reported to be very good (Cohen's kappa ranging between κ = 0.85 and κ = 0.98). Semantic segmentation algorithms can be used to detect shapes that are clearly distinguishable from the background. However, for studying human interactions, the areas of interest are not necessarily distinguishable from one another by a semantic border. For example, there is no visually distinctive line separating the areas of interest “chest” and “pelvis.” In the case of human interactions, semantic segmentation algorithms alone are not sufficient to differentiate between the desired areas of interest.

To solve the issue, we propose the use of an additional algorithm, namely an *instance segmentation algorithm* that can be used in tandem to differentiate between areas of interest. This algorithm can locate the center point of a specific area of interest in an image. Prominent instance segmentation algorithms such as OpenPose (Cao et al., 2016) have been successfully applied to track facial landmarks (Fujiwara & Yokomitsu, 2021; Hessels et al., 2018) and body joints (De Beugher et al., 2018) in video footage. An instance segmentation algorithm is trained to identify a pixel coordinate (*x* and *y*) in an image, where the pixel corresponds best to a single location. When trained to identify human body locations, such as the hand, the algorithm returns the pixel coordinates at the location that best represents the body location. The coordinates of each identified body location can then be compared to the gaze coordinates in order to determine which body location the gaze is closest to.

The aim of this study was to examine the extent to which the footage obtained using mobile eye-tracking in a fast-paced human interaction task could be annotated automatically using computer vision algorithms. We recorded eye-tracking footage during a series of grip fights, with the aim of automatically analyzing the gaze footage using a combination of the semantic segmentation and instance segmentation algorithms. We tested the algorithms by comparing their performance against the performance of three manual raters. If successful, the algorithm offers promise for the fast and objective analysis of mobile eye-tracking data.

## Method

### Task design

Eight judokas participated as part of a broader study examining visual behavior in combat sports (Krabben et al., 2022). The experimental task consisted of a series of short judo grip-fighting sequences. The grip fight represents a complex human interactive task where the two judokas try to obtain an advantageous grip on their opponent while simultaneously avoiding being gripped by that opponent. Typical to the sport of Judo, two participants started by facing each other approximately 2.5 m apart and then, after a verbal signal from the researcher, competed to establish a two-handed grip on their opponent’s jacket. The task stopped when one of the participants was able to establish a firm grip, after which the researcher verbally instructed the participants to move back to their respective starting positions. In contrast to regular Judo competition, the participants were interacting with their opponent using only their hands, and not their feet. The grip-fighting task was designed to examine the gaze strategies deployed during the grip fight, where visual information about the opponent’s movements is crucial for success. Participants were free to move in a designated area of 3 × 3 m during the grip fighting.

### Task procedure

The judokas were placed into pairs for the grip-fighting task and competed in two sets of 20 grip-fight exchanges. In each of the sets, one participant was fitted with an eye tracker (Pupil Core; Pupil Labs, Berlin, Germany; Kassner et al., 2014), with the eye tracker transferred to the other participant for the second set. The eye tracker was calibrated before each set and after every fifth trial. The calibration locations were five circles with a diameter of approximately 12 cm printed on paper and fixed to a wall, spanning 2 × 2 m in the shape of a plus sign, with one marker placed in the middle. For the calibration, the participant stood 2 m from the wall and was asked to move their eyes to fixate the markers in a predefined order. After the calibration, the participants were asked to direct their gaze towards one of the markers to check whether the eye tracker was successfully calibrated. The distance between the participant and the wall and the width of the marker were both taken into account to assess the accuracy of the calibration. If the gaze was adjacent to/outside of the marker (indicating the calibration error was greater than 3.5°), the calibration was repeated. Then, participants engaged in the 20 grip-fighting sequences. The sequences were initiated by an audio signal (one of the participants clapping their hands) and ended when one judoka was able to perform a grip on the opponent. The footage was collected from the world camera view of the eye tracker and cropped to the start and end moments for analysis. The cropped sequence was exported using Pupil Player software (Kassner et al., 2014). In the exported footage, the combined binocular gaze position was calculated and visualized as a singular point on the exported video. The Pupil Player software exported the world camera footage as a video file, and the gaze positions as comma-separated values for each frame of the footage.

### Dataset

The data consisted of samples taken from randomly selected trials from the dataset of Krabben et al. (2022). The dataset consisted of 1,188 frames of footage, sampled from 13 trials with six different participants appearing in the trials. We made the deliberate choice to take a representative sample that contained footage from several participants exhibiting in situ behavior. We made this choice acknowledging that the gaze distribution would likely not be equally distributed between areas of interest, because humans frequently use gaze anchors in such tasks (Vater et al., 2020; Williams & Elliott, 1999), which might lead to an overrepresentation of some areas of interest compared to other areas of interest. As a consequence, an appropriate statistical analysis needed to be undertaken to address the imbalance in the samples. The strength of the sample taken is that it allows us to infer to what extent the proposed algorithm would be able to match the annotations of manual raters in a dynamic real-life task. The same data that were analyzed by the manual raters were reanalyzed using the proposed computer vision algorithm for the purpose of comparison.

### Algorithmic analysis

For the algorithmic analysis, the input for the algorithm consisted of two parts: the exported footage from the world camera (video file) and the frame-by-frame gaze locations. The results of the algorithmic analysis were exported to a comma-separated text file for statistical analysis. In this method, the algorithm generated a fully automated annotation of the direction of gaze on each frame by comparing the gaze direction in relation to one of the seven prespecified areas of interest recognized by the algorithms (semantic regions with the shape of a human).

**Fig. 1:**
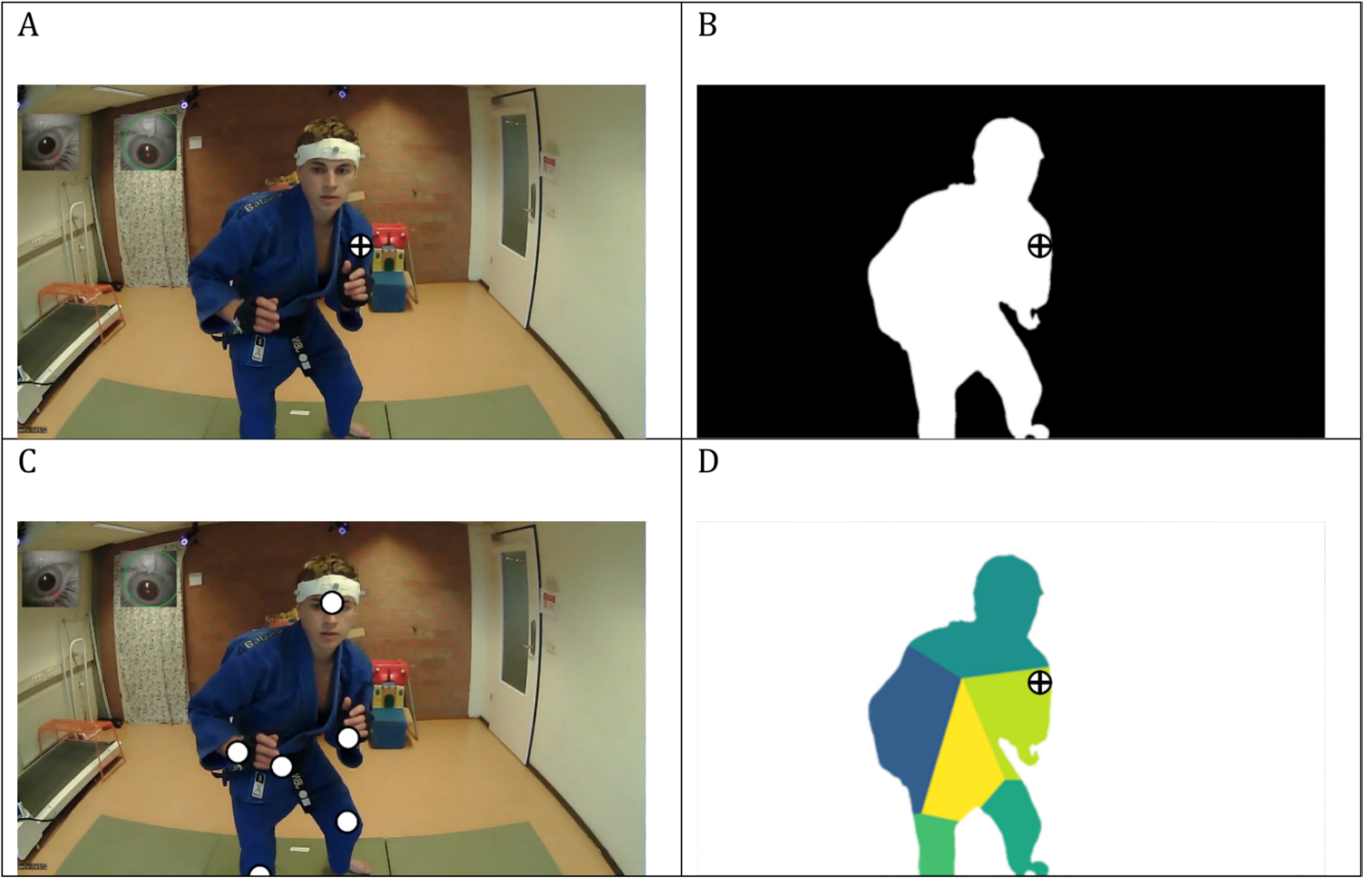
Algorithmic annotation of eye-tracking data. **A** The input consists of an image from the world view and the gaze location, here a black cross. **B** In a first step, the human is identified using a semantic segmentation, Mask R-CNN (He et al., 2017) **C** Body locations are identified on the human using an instance segmentation algorithm, OpenPose (Cao et al., 2016). **D** The human shape (from **B**) is divided into areas of interest based on a selection of body locations (from **C**). The gaze overlay indicates that the gaze is inside of the human shape, directed at “left arm,” and is annotated by the algorithm accordingly

The input to the algorithmic approach consisted of the world video of the eye tracker, together with the *x*- and *y*-coordinates of the binocular gaze point (Fig. 1A). In a first step, the semantic segmentation algorithm was applied to each frame of the video footage (He et al., 2017). The algorithm detects areas in the image that are associated with the shape of a human. All other areas are masked (Fig. 1B). Then, to identify areas of interest in the footage, the instance segmentation algorithm was applied to the same video footage. The OpenPose algorithm (Cao et al., 2016) picks up on 25 predefined joint centers (Fig. 1C). To compute the areas of interest, we selected a subset of seven centers from the available 25 centers to proceed (the center location of the head, chest, pelvis, right wrist, left wrist, right ankle, and left ankle were selected). Those joint centers served as centroids for each corresponding area of interest. Finally (Fig. 1D), the results of the two algorithms were combined. The human shape identified by the semantic segmentation algorithm was divided into seven regions using a Voronoi tessellation, each representing an area of interest. For each pixel within the human form, the closest of the seven body markers was identified, and the pixel colored accordingly. The location of gaze was then compared against this colored map. In Fig. 1, the annotation was marked as belonging to the “left arm” category, indicating that the gaze was directed towards one of the areas of interest.

### Manual analysis

To assess the quality of the proposed algorithm, three manual raters with experience analyzing mobile eye-tracking data viewed a subset of the collected eye-tracking data from the perspective of one of the Judokas. The video footage of the world view from the eye tracker was presented in a custom web user interface with the gaze location overlaid as a dot. The manual raters were required to annotate for each frame whether gaze was directed towards the opponent, and if so, which of the seven areas of interest gaze was most likely directed towards. The instructions were provided in written form on the web interface: “Annotate for each frame where the gaze is directed to and tick the corresponding area of interest accordingly. In case you find that the gaze was not directed at one of the areas of interest, tick the ‘other’ category.” The manual annotators were not given explicit instructions regarding boundaries or rules that helped them identify areas of interest. We chose this approach because it aligns with previous studies analyzing eye-tracking footage by manual raters where, to the best of our knowledge, no specific instructions were provided to the raters regarding how to determine the boundaries for the areas of interest (Piras et al., 2014; Spitz et al., 2016; Van Biemen et al., 2023). We acknowledge that more detailed instructions could potentially improve the inter- and intra-rater reliability, but we chose to follow what we felt best reflected previous approaches. The raters were able to move back and forth between frames where needed. The order of presentation of the trials was randomized, and all manual raters were presented with the same randomized order. The annotations of the manual raters were saved to a file and analyzed using Python (Rossum & Drake, 2010) and the Krippendorff package (Castro, 2017).

### Data analysis

The ratings of the manual raters served as a ground truth against which the algorithm was compared. The ground truth was established for each frame if at least two of the three raters agreed in their judgment. In the rare case where all three human raters came to a different conclusion, no ground truth was established, and the frame was discarded from the analysis. For all the frames where at least two raters agreed, the majority vote was used to define the ground truth label, and it was against that ground truth that the algorithm was compared. Two analyses were used in tandem to examine the ratings.

First, the agreement among the expert raters was assessed using Krippendorff’s alpha (Hayes & Krippendorff, 2007). Then, the same statistic was used to assess the overall agreement of the raters and the algorithm. Following the convention of Cohen (1960), effect sizes less than 0.2 were treated as small, 0.2 to 0.4 as moderate, 0.4 to 0.6 as substantial, and greater than 0.8 as large.

Second, to assess the accuracy of the annotations, the F-measure (Hripcsak & Rothschild, 2005) was computed for each area of interest. The F-measure is a signal detection metric composed of two components: *precision* and *recall*. Precision is the percentage of true positives out of all positives predicted by the model. In other words, precision answers the question, “Of all the instances the algorithm labeled as positive, how many were actually positive?” Recall, on the other hand, is the percentage of true positives as identified by the raters out of all actual positives in the data. It addresses the question, “Of all the actual positive instances in the data, how many did the algorithm successfully identify?” Precision and recall are commonly used in computer vision research to quantify classification algorithms for samples with skewed distributions (Powers, 2020). The F-measure returns a value between 0 and 1, where higher values indicate better performance. In the case of a well-performing algorithm, both precision and recall should be balanced and high. To calculate the F-measure, the precision and recall values are combined into a single metric using the harmonic mean of precision and recall.

## Results

### Algorithmic rating

**Fig. 2:**
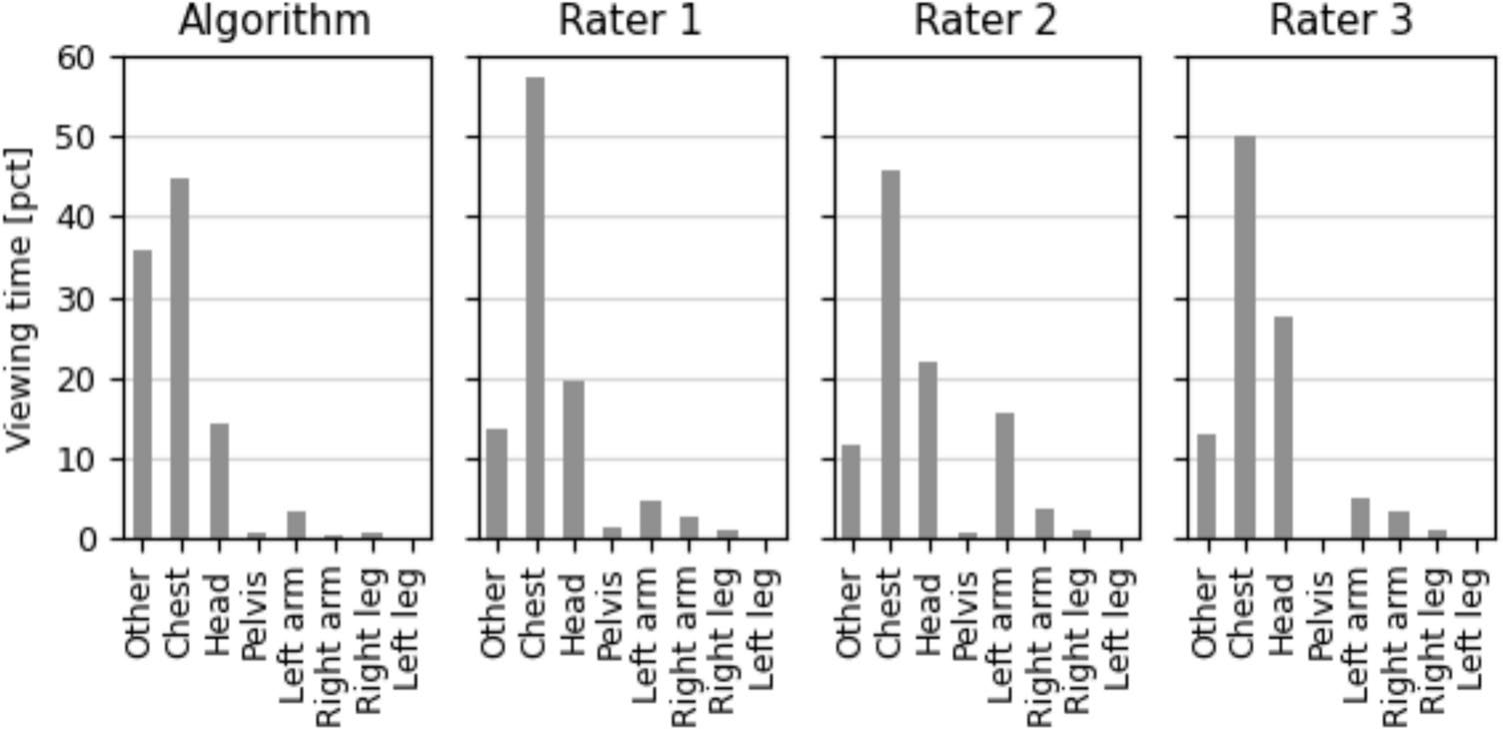
Percent viewing time for areas of interest according to the algorithmic annotation and each individual human rater

The algorithm annotated each frame in approximately 0.35 s, with the time approximately split as follows: semantic segmentation consumed 0.2 s and instance segmentation took 0.1 s. For the analyzed frames, the algorithm was able to identify the human in every frame (100%). According to the algorithmic annotation (Fig. 2), gaze was directed most frequently towards the chest (44.8% of frames), followed by the head (14.3%), left arm (3.28%), right leg (0.76%), pelvis (0.76%), right arm (0.25%), and left leg (0%). In 35.9% of the frames, the algorithm annotated the area as “other.”

### Human ratings

We compared the ratings among the three manual raters for the annotations of gaze. All three raters annotated the same area of interest in 68.3% of the frames, meaning that the agreement between the manual raters was not perfect for about one third (*n* = 376) of the frames. In a small percentage of the frames (*n* = 34), each rater allocated a different area of interest, and these frames were discarded from the analysis. This means that at least two of the three raters agreed on 97.1% of the frames. The agreement between the human raters was κ = 0.67 using Krippendorff’s alpha, which can be interpreted as a substantial level of agreement (Landis & Koch, 1977). According to the manual raters (Fig. 2), gaze was directed predominantly towards the chest of the opponent (52.3% of frames), followed by the opponent’s head (27.0%), left arm (3.6%), right arm (2.0%), right leg (1.2%), and left leg (0%). The human raters annotated 13.9% of frames to the category “other.”

### Agreement between human raters and the algorithm

**Fig. 3:**
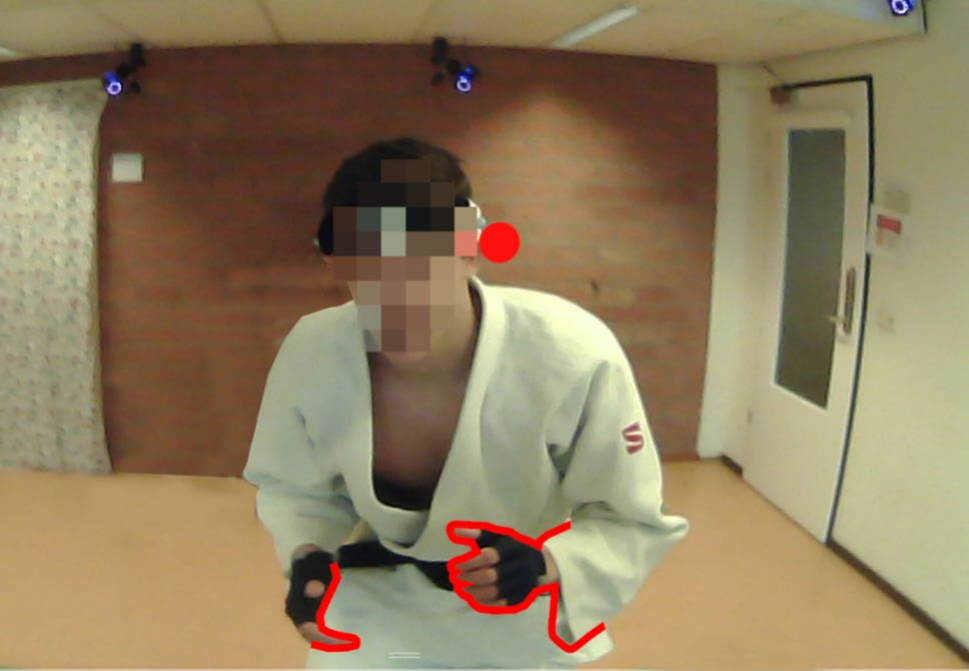
Frame with gaze indicator (red circle) just outside of the head region

The interrater agreement between the algorithm and the human raters was moderate (Krippendorff’s alpha = 0.49) across all frames. The most notable difference between the annotations of the algorithm and the human raters was that the algorithm annotated a greater portion of frames to the area of interest “other” than the manual raters (35.9% of the frames vs. 13.9% on average, respectively). Visual inspection of the footage revealed many instances of frames where gaze in the eye-tracking footage was located just outside of the human body (see Fig. 3 for an example). The semantic segmentation algorithm tagged those frames as “other,” while human raters interpreted gaze as being directed towards their opponent. When analyzing only the areas of interest that were located on the opponent’s body—and therefore leaving out the “other” area of interest—the agreement improved and was substantial (Krippendorff’s alpha = 0.61).

**Fig. 4:**
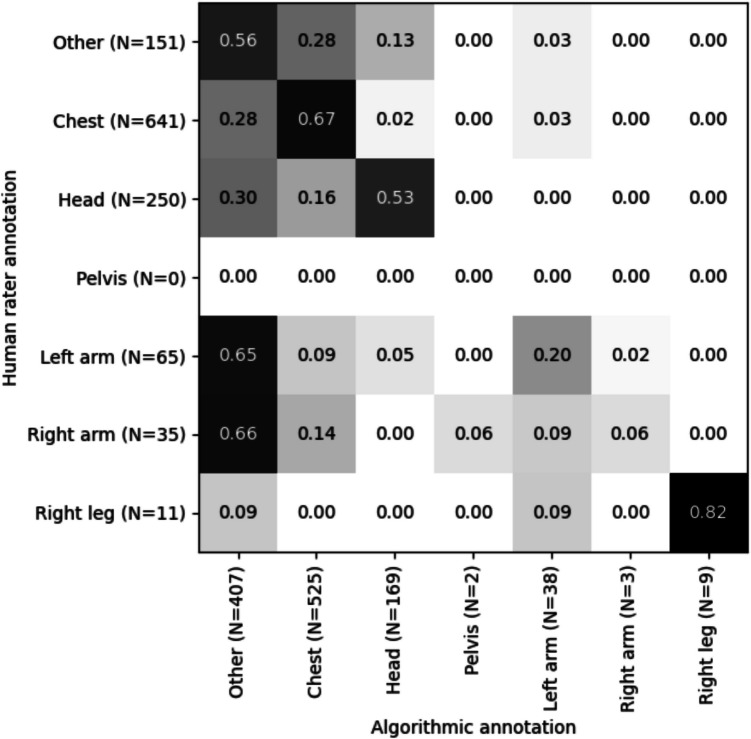
Normalized confusion matrix for human versus algorithmic annotations in the interactive grip-fighting task. The matrix compares the frequency of annotations for different anatomical regions by human raters (rows) against those by the automated algorithm (columns). Each cell represents the proportion of total annotations for that row, with the sum of each row equal to 1. Regions with higher agreement between the algorithm and the human raters are highlighted with darker shades

For each of the areas of interest, the agreement between the human raters and the algorithm is shown in the form of a confusion matrix in Fig. 4, a visualization frequently used in the computer vision literature to discuss the accuracy of an algorithm when compared to validation ratings. The confusion matrix reveals on the diagonal (top left to bottom right) to what extent the algorithm and the human raters have annotated a frame to the same area of interest, with high agreement reflected in higher numbers (values closer to 1) and lower agreement indicating lower agreement (values closer to 0). The takeaway from Fig. 4 is that there is that there is a general pattern of accordance between the algorithm and the raters along the diagonal line. The exception to this pattern is high values in the bottom left of the figure, in the first column. This column describes cases where the algorithm annotated a frame as “other” and reveals the areas that the human raters annotated for the same frame. Notably, the takeaway is that human raters annotate frames differently in cases when the algorithm annotates the same frames as “other.”

Given that gaze was not normally distributed across the seven areas of interest (i.e., the distribution was skewed), we computed F-measure statistics for each of the areas of interest to account for the skewed distribution and to compare the accuracy for each area individually (Table 1).

**Table 1:** F-scores for rating agreement

AOI	Precision	Recall	F1-score
Other	0.21	0.56	0.30
Chest	0.82	0.67	0.74
Head	0.79	0.53	0.63
Pelvis	0.00	0.00	0.00
Left arm	0.34	0.20	0.25
Right arm	0.67	0.06	0.11
Right leg	1.00	0.82	0.90

The algorithm annotated 407 frames as belonging to the area “other,” while the human raters were much more conservative and annotated only 151 frames as “other.” The difference is reflected in the low precision score of 0.21. Despite the low precision, the recall is relatively high at 0.56, indicating that the algorithm is capturing many true “other” instances. The F1 score of 0.30 suggests that the low precision score undermines the algorithm’s ability to pick up the area “other” consistently.

The algorithm annotated 525 frames as belonging to the chest, and for the majority of these frames, the annotations were consistent with the ratings of the human raters, evidenced by a precision score of 0.82. The confusion matrix highlights the algorithm's tendency to mistakenly label chest as “other” (28% of human annotations were mislabeled), as head (16%), as right arm (14%), or as left arm (9%). Human raters identified the chest area in 641 instances, more often than the algorithm, leading to a lower recall score of 0.67. This suggests that while the algorithm is reliable when it identifies the chest, it is not as effective at detecting all instances. The combined F1 score for chest is 0.74, which was the second-highest scoring category in the analyzed dataset.

The algorithm identified 169 frames as being towards the Head, with a precision score of 0.79. The precision indicates a high likelihood that the algorithm's annotations are correct. However, the human raters labeled Head more frequently, in 250 instances, revealing that the algorithm missed several true Head annotations, as shown by a recall of 0.53. This discrepancy, as illustrated by the confusion matrix, indicates that the algorithm may confuse head with “other.” Since both precision and recall are relatively balanced, the head area results in the third-highest F1 score.

The pelvis category was not annotated by the human raters, resulting in zero instances where the algorithm could have potentially agreed or disagreed (F1 score of 0.00).

The algorithm's performance on the left arm was marked by low precision and recall, with only 38 frames correctly annotated, resulting in precision of 0.34. The human raters applied the left arm label 65 times, but the algorithm failed to recognize many of these, leading to recall of 0.20. For the right arm, the algorithm annotated only three frames, mostly correctly (precision = 0.67), but failed to identify most instances when the humans annotated a frame as right arm (recall = 0.06). Human raters labeled the right arm 35 times, and the algorithm missed most of these true instances. Due to the low recall, the overall F1 score was 0.11.

In the few instances where the algorithm annotated an area as belonging to the right leg, the annotation was mostly on point, resulting in an F1 score of 0.90. The algorithm annotated the right leg with perfect precision (1.00) and high recall (0.82).

The left leg was not annotated on any frame by either the human raters or the algorithm, and no results were calculated for this area of interest.

The overall F1 score calculated across areas of interest was 0.61 (precision = 0.70, recall = 0.58) for the frames where at least two raters agreed. We also calculated the F1 score post hoc for only the samples where all three raters agreed, and the score improved marginally to 0.64 (precision = 0.70, recall = 0.61). It appears that the algorithm performs slightly better for frames where the manual raters had total agreement, and the improvement seems to be linked to the improved recall rate, which indicates that the algorithmic labels were more likely to detect the annotations made by the manual raters.

## Discussion

This study was designed to examine the extent to which mobile eye-tracking footage in an interactive human task could be annotated automatically by computer vision algorithms. A combination of two computer vision algorithms was used to annotate gaze in an automated fashion in an interactive sports task. The critical question was whether an algorithm could identify fast-moving athletes and segment the dynamic areas of interest as reliably as expert manual raters could. To validate the annotations of the algorithm, expert manual raters annotated the same footage to determine whether gaze was directed towards one of seven predefined areas of interest. The main finding of this study was that the computer vision algorithm was able to annotate the fast-moving areas of interest as reliably as the manual raters (Krippendorff’s alpha = 0.67 between human raters and 0.61 between human raters and the algorithm). But the agreement was less clear when gaze was directed outside of the opponent’s body. In that case, when the category “other” was included, the agreement between the human raters and the algorithm was lower but still moderate (Krippendorff’s alpha = 0.49).

The algorithmic approach annotated more frames to the area “other” than any of the human raters. The most likely explanation is that human raters use a different annotation strategy than the algorithm when gaze is directed just outside of the human opponent (as illustrated in Fig. 3). When the gaze indicator is outside of the human opponent for just a few consecutive frames, human raters seem to be more lenient and tend to annotate the gaze to the human body. In other words, it looks as though human raters, for any given frame of video footage, do not code gaze independently of the other frames. Instead, the human raters view the frames in sequence and know where the gaze was prior to that frame, and can look to see where the gaze was on subsequent frames. Moreover, human raters know that there is noise in the gaze signal and therefore that it will deviate to some degree away from where it is likely directed. For instance, the indicated gaze position can be perturbed by the movement of the wearer, shifted due to slippage, or calibrated imprecisely. With that information in mind, humans may interpret gaze on some given frames as being towards a body part, particularly on frames where it has momentarily moved away from the body. The algorithm, however, does not take this contextual information into account, and so it will code gaze as being outside of the body in any instance where that occurs. The algorithm, in that sense, is not attuned to making use of this contextual information but defines the area of interest strictly along the semantic borders of the footage.

The remaining disagreements between the algorithm and the raters were likely linked to areas of interest that were partially occluded by another area of interest. As described in previous research on combat fighting (Hausegger et al., 2019; Piras et al., 2014), gaze is frequently directed towards the chest area of the opponent while fighting, which provides a stable gaze anchor (Vater et al., 2020). Given the gaze anchor, there are moments, however, where for instance the hands moved dynamically in front of the chest. In those frames, both areas of interest overlap, and the algorithmic approach annotates the frame without contextual knowledge of the previous and following frames and simply allocates gaze to the most proximal body part visible from the participant’s point of view. Human raters, on the other hand, may again take contextual information into account (i.e., where gaze was on previous and future frames) and ignore an area of interest that temporally occludes another.

To validate the algorithmic approach, the annotations of the algorithms were compared to the annotations made by human raters. For the sake of the validation, the annotations of the human raters served as a ground truth, although there are doubts about the degree to which human classification can be considered a “gold standard” (Hooge et al., 2018). In our study, all three human raters agreed in only about two thirds of the frames to which area of interest gaze was supposedly directed. The problem of reaching an agreement is likely to increase when the raters must judge between areas of interest that do not have clear borders. For instance, in studies of human interactions, the body regions (e.g., chest, pelvis, leg) are often occluded behind clothing. The occlusion makes it hard to define rules for gaze annotation, which ultimately leads to lower agreement between human raters. In this study, the manual raters were not provided with specific rules that helped them to mitigate some of these concerns. The reason that no explicit rules were provided in the instructions is that several sources of error can be at play at the same time, which would require a complex set of rules to be hand-crafted for the specific task. For instance, the gaze indicator can be perturbed due to slippage, plus a shift due to a calibration artifact, plus uncertainty arising from an occluded area of interest. We took the deliberate choice of not attempting to define specific rules for these edge cases, but instead let the raters come to their conclusion based on their experience analyzing such data. A similar approach has been used by other studies analyzing human interactions in realistic settings (Hausegger et al., 2019; Piras et al., 2014).

Computer vision algorithms offer a distinct advantage over manual annotation, because the analysis is fast and repeatable, and will return the same results when executed again. For conducting research, the possibility of speeding up the annotation process in particular can allow researchers to analyze larger and more complex datasets to produce insights about human interactions (Kredel et al., 2023). In this study, like the manual raters, the computer vision algorithm was not explicitly tuned to incorporate temporal aspects into the analysis, in order to create a fair comparison.

There are at least two potential solutions to the possible shortcomings of the algorithms we implemented in our study. First, to address the discrepancy with which the area “other” was tagged, an additional rule could be added to the algorithm to make the algorithm annotate gaze to the body even in cases when the gaze direction lies just outside the human body shape. For instance, Hessels et al. (2016) discuss the robustness of eye-tracking annotations when areas of interest are enlarged prior to the analysis. In particular, the authors note that Voronoi-based approaches create an objective way to analyze the data. In their analysis, enlarging the Voronoi-based areas of interest by up to 3° of visual angle enhanced the robustness of eye-tracking in their study of facial expressions. For our solution, the distance from the gaze to the nearest body part could be calculated and used as a threshold for adding leniency, such that a frame would be annotated to the body as long as the gaze direction was within the defined threshold. Second, the algorithm does not preserve any temporal information and annotates each frame by itself. In both the case of gaze being temporarily directed outside of the body and the case of temporal overlap of areas of interest, a temporal smoothing approach could be applied to filter out any occurrences where the gaze was, for only a single or a few frames, directed at an area of interest, while being directed at another area of interest before and after the temporal occlusion.

The promise of the computer vision algorithms is that a combination of semantic segmentation and instance segmentation can be used to identify areas of interest, such as a human body that can be further divided into body parts in an objective time-saving and deterministic manner. The findings from recent studies employing computer vision to facilitate the analysis of eye-tracking data were extended, as this study demonstrates the efficacy of an algorithmic approach even for fast-paced actions that are common in natural human interactions in sport.

## Data Availability

The anonymized data and the analysis scripts needed to replicate the findings of this study are retrievable via 10.5281/zenodo.13832500.

## Code availability

The open-source Python package is available under https://github.com/footballdaniel/gazeclassify and via the Python package installer PyPi https://pypi.org/project/gazeclassify/.
